# Short-term effects and safety of an acute increase of intraocular pressure after intravitreal bevacizumab injection on corneal endothelial cells

**DOI:** 10.1186/s12886-018-0682-9

**Published:** 2018-01-25

**Authors:** Jiwoong Park, Myungwon Lee

**Affiliations:** Department of Ophthalmology, College of Medicine, Dankook University, 119, Dandae-ro, Dnognam-gu, Cheonan-si, Chungchungnam-do Republic of Korea

**Keywords:** Intraocular pressure, Intravitreal injection, Bevacizumab, Corneal endothelial cells, Specula microscopy

## Abstract

**Background:**

The purpose of this study is to evaluate short-term effects and safety of an acute increase of intraocular pressure (IOP) after single-dose intravitreal bevacizumab injection on corneal endothelial cells and central corneal thickness.

**Methods:**

Forty-two patients who underwent intravitreal injection of 2.5 mg/0.1 ml bevacizumab because of central serous chorioretinopathy or diabetic macular edema were included in this study. The changes of IOP, corneal endothelial cells, and corneal thickness at baseline, 2 min, 5 min, and 30 min after injection were analyzed prospectively with a specular microscope.

**Results:**

In all patients, the mean IOPs at baseline, 2 min, 5 min, and 30 min after injection were 11.48 ± 2.22 mmHg, 49.71 ± 10.73 mmHg, 37.64 ± 11.68 mmHg, and 14.88 ± 4.77 mmHg, respectively. These changes were significant (*p* < 0.01). In only one eye, IOP did not decrease to ≤30 mmHg even at 30 min after injection. According to changes in IOP with time, the coefficient of variation of the corneal endothelium significantly increased (*p* = 0.03), but cell density, hexagonality of the corneal endothelium, and central corneal thickness did not change (*p* = 0.79, 0.21, and 0.08, prospectively). One week after injection, there was no sign of inflammation or any other complications in all 42 eyes.

**Conclusions:**

After intravitreal injection, IOP rapidly increases, then decreases to the normal range in most eyes 30 min after injection and it is tolerable to corneal endothelium.

**Trial registration:**

Clinical Research Information Service (CRiS), Republic of Korea, KCT0002645. Retrospectively registered 9 January 2018.

## Background

Corneal endothelial cells constitute the innermost layer of the cornea, where they contribute to structural integrity. The sodium-potassium pump of endothelial cells plays an important role in maintaining the transparency and dehydrated state of the cornea [1]. It has been reported that once corneal endothelial cells are damaged, they cannot be regenerated [2], but can be complemented by the expansion of perimetric normal endothelial cells, the fusion between cells, or in rare cases, by cell division [3, 4]. Increased intraocular pressure (IOP) due to various reasons causes corneal edema and damage to the endothelial cells with optic nerve damage, and can lead to significant loss of vision.

IOP increases when therapeutic intravitreal bevacizumab (Avastin®; Genentech, South San Francisco, CA, USA) injection without anterior chamber paracentesis is performed [5]. Although intravitreal bevacizumab injection results in increased IOP, at 30 min after injection, Judy et al. [6] reported that the IOP dropped to a normal range of < 30 mmHg in all patients without anterior chamber paracentesis. Benz et al. [7] also reported that the IOP dropped to normal levels 30 min after injection in almost all patients. In an in vivo study, Chiang and associates [8] demonstrated that there are no harmful effects on the corneal endothelial cells after a standard intravitreal bevacizumab injection. Based on these reports, this study prospectively evaluated momentary changes and safety of corneal endothelial cells and corneal thickness immediately after an acute increase of IOP resulting from therapeutic intravitreal injection of bevacizumab.

## Methods

We selected 42 eyes of 42 patients who were treated with intravitreal bevacizumab injection due to macula edema or macular degeneration between May 2014 and January 2015 at Dankook University Hospital, Cheonan, South Korea. This evaluation was performed prospectively, and approval was obtained from the Dankook University Hospital Institutional Review Board, Cheonan, South Korea. All procedures followed the Declaration of Helsinki rules and written informed consent was obtained from all patients.

The causes of macular edema included diabetic macular edema, retinal vein occlusion (central, branch), and central serous chorioretinopathy (Table 1). Patients were excluded if they had any ocular disease, previous any intravitreal injection, use of topical eyedrops for lowering IOP, or any ocular surgery history except cataract surgery that had no complication like phacoemulsification and posterior chamber lens implantation (PE&PCL). All 42 contralateral eyes had no history of intravitreal injection and were used as the control group. The patients were grouped by diabetes mellitus (DM) and a history of cataract surgery.

**Table 1:** Characteristic of Eyes Undergoing Intravitreal Bevacizumab Injection

Parameter	
Age (years, mean ± SD^*^)	57.81 ± 14.11
Sex (M/F)	18/24
Diabetes mellitus	13 (31%)
Diagnosis (eyes)	
BRVO	11 (26.2%)
AMD	10 (23.8%)
PDR	7 (16.7%)
CRVO	6 (14.3%)
CSC	5 (11.9%)
DME	3 (7.1%)
Surgical history (eyes)	
Cataract surgery	5 (11.9%)

*M* male, *F* female, *SD* standard deviation, *BRVO* branch retinal vein occlusion, *AMD* age related macular degeneration, *PDR* proliferative diabetic retinopathy, *CRVO* central retinal vein occlusion, *CSC* central serous chorioretinopathy, *DME* diabetic macular edema

All intravitreal bevacizumab injections were performed under aseptic conditions using a surgical microscope. The patient eye was anesthetized using 0.5% topical proparacaine hydrochloride (Alcain®; Alcon, Fort Worth, TX, USA). Bevacizumab at 2.5 mg/0.1 mL was injected into the vitreous with a 30-gauge needle through the supratemporal pars plana at 3.0 mm or 3.5 mm posterior to the limbus if the patient had a pseudophakic or phakic eye, respectively. Based on a previous report that the IOP decreases to a normal range without anterior chamber paracentesis after 30 min [5, 9], anterior chamber paracentesis was not performed. After intravitreal injection, the patient used fourth generation quinolone topical drugs, and after 1 week, evaluation of inflammation was performed using slit lamp microscopy.

Before injection, patients underwent a complete ophthalmic examination including visual acuity tests with and without correction, slit-lamp examination, fundus examination, and optical coherence tomography (Cirrus HD-OCT®, version 5; Carl Zeiss Meditec, Dublin, CA, USA). IOP and endothelial cell evaluations were performed before injection, and 2 min, 5 min, and 30 min after injection. The area and density of endothelial cells and the central corneal thickness (CCT) were evaluated by non-contact specular microscopy (SP-3000P; Topcon, Tokyo, Japan). To minimize evaluation bias, two measurements were made in the center of the cornea to obtain a mean value. If the boundary of endothelial cells was unclear or not well focused, remeasurements were performed. To prevent infection, IOP was not evaluated by Goldmann applanation tonometry, but by a non-contact tonometer (CT-80; Topcon). IOP was measured three times and the mean value was used. If the non-contact tonometer could not measure the IOP, rebound tonometry (ICare™; Finland Oy, Vanda, Finland) was used with a new tip at each measurement. Between each measurement, topical antibiotics were used once, and the patients were instructed not to expose the treated eye. After the last follow-up during the 30 min period, a patch with ofloxacin ointment was applied to the patient’s eye.

Using a built-in program in the non-contact specular microscope, the cell density (CD; cells/mm^2^), coefficient of variation of the cell area (CV; polymegathism), and hexagonality (6A; polymorphism) of the endothelium were evaluated. Changes were expressed as the mean ± standard deviation.

### Statistical analysis

Statistical analyses were performed using SPSS Statistics software for Windows, version 18.0 (SPSS, Chicago, IL, USA). One-way ANOVA test was used to compare parameters between the affected and control groups. Mann-whitney U test was used as a nonparametric test to determine the difference in parameters between patients with and without DM. It was also used between phakic and pseudophakic eyes. One-way repeated measures ANOVA, Bonferroni correction were used to observe whether the IOP and measured values change significantly over time. Linear mixed model was used for changes of corneal endothelial cells and CCT after intravitreal injection, by determining correlations between measurements of the same patient. A *p*-value < 0.05 was considered significant.

## Results

The study included a total of 42 eyes from 42 patients, including 18 males and 24 females. The mean age was 57.81 ± 14.11 years. Eleven eyes (26.2%) with branch retinal vein occlusion (BRVO), 10 eyes (23.8%) with age-related macular degeneration (AMD), and 7 eyes (16.7%) with proliferative diabetic retinopathy (PDR) accounted for 66.7% of all patients. The other eyes included 6 eyes (14.3%) with central retinal vein occlusion (CRVO), 5 eyes (11.9%) with central serous chorioretinopathy, and 3 eyes (7.1%) with diabetic macular edema (DME). All 42 eyes were injected for the first time. There were 13 eyes (31%) with DM, and 5 eyes (11%) with a history of cataract surgery (Table 1).

In all patients, The CD before injection were 2605.01 ± 399.08 cells/mm^2^ in the treated group, and 2583.90 ± 377.20 cells/mm^2^ in the normal control group. There was no significant difference between the groups (*p* = 0.80). The CV and 6A of the endothelium, and the CCT also showed no significant difference between the groups (*p* = 0.36, 0.78, 0.91) (Table 2).

**Table 2:** Comparisons between the treated Group and the Normal Control Group

	Treated (*n* = 42)	Control (*n* = 42)	*p*-value^a^
IOP(mmHg)	11.48 ± 2.22	12.64 ± 3.81	0.09
CD(cells/mm^2^)	2605.01 ± 399.08	2583.90 ± 377.20	0.80
CV	64.65 ± 32.90	71.75 ± 37.47	0.36
6A(%)	39.95 ± 10.77	40.71 ± 13.52	0.78
CCT(μm)	508.01 ± 32.54	507.21 ± 31.56	0.91

*IOP* intraocular pressure, *CD* corneal density, *CV* coefficient of variation, *6A* hexagonality, *CCT* central corneal thickness

^a^One-way ANOVA test

Patients were divided into two groups based upon a diagnosis of DM. The CD was 2557.57 ± 403.07 cells/mm^2^ in non-DM patients (*n* = 29) and 2710.83 ± 384.08 cells/mm^2^ in DM patients (*n* = 13), which didn’t show significant difference (*p* = 0.37). The other parameters, including IOP, also showed no significant difference (Table 3).

**Table 3:** Comparisons between Patients with or without DM

	With DM (*n* = 13)	Without DM (*n* = 29)	*p*-value^a^
IOP(mmHg)	11.76 ± 2.35	11.34 ± 2.19	0.65
CD(cells/mm^2^)	2710.83 ± 384.08	2557.57 ± 403.07	0.37
CV	57.93 ± 11.76	67.66 ± 38.67	0.91
6A(%)	41.00 ± 7.76	39.48 ± 11.97	0.67
CCT(μm)	507.92 ± 33.17	508.03 ± 32.84	0.96

*DM* diabetes mellitus, *IOP* intraocular pressure, *CD* corneal density, *CV* coefficient of variation, *6A* hexagonality, *CCT* central corneal thickness

^a^Mann-whitney U test

The mean age of patients with or without a history of cataract surgery (pseudophakic or phakic eyes) were 55.16 ± 12.83 years, 77.40 ± 4.28 years, and CD were 2098.88 ± 181.83 cells/mm2, 2673.41 ± 370.58 cells/mm2, respectively. Age was older and CD was lower in pseudophakic eyes, which were both statistically significant (*p* < 0.01, *p* < 0.01) (Table 4).

**Table 4:** Comparisons between phakic and pseudophakic eyes

	Phakic eyes (*n* = 37)	Pseudophakic eyes (*n* = 5)	*p*-value^a^
Age(years)	55.16 ± 12.83	77.40 ± 4.28	< 0.01
IOP(mmHg)	11.57 ± 2.27	10.80 ± 1.92	0.50
CD(cells/mm^2^)	2673.41 ± 370.58	2098.88 ± 181.83	< 0.01
CV	64.05 ± 31.80	69.14 ± 44.42	0.60
6A(%)	40.30 ± 10.29	37.40 ± 15.08	0.82
CCT(μm)	509.00 ± 33.93	500.60 ± 20.45	0.74

*IOP* intraocular pressure, *CD* corneal density, *CV* coefficient of variation, *6A* hexagonality, *CCT* central corneal thickness

^a^Mann-whitney U test

**Fig. 1:**
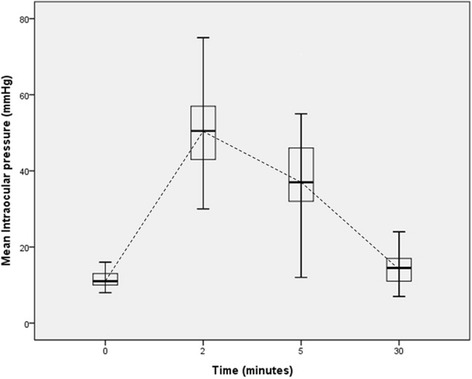
Intraocular pressure changes after intravitreal bevacizumab injections. Intraocular pressure increased after 2 and 5 min in a stepwise manner, and returned to a normal range after 30 min

In the treated group, the IOP at baseline was 11.48 ± 2.22 mmHg. At 2 min and 5 min after injection, it was 49.71 ± 10.73 mmHg and 37.64 ± 11.68 mmHg, respectively, and showed a significant increase. Thirty minutes after injection, it returned to a normal range (mean value, 14.88 ± 4.77 mmHg). In only one eye, the IOP did not decrease by 30 mmHg or less, even after 30 min (31 mmHg), but after 1 h, it returned to a normal range (19 mmHg; Table 4). Before injection, and 2 min and 5 min after injection, the IOP significantly increased in a stepwise manner (all, *p* < 0.01). At 30 min after injection, it decreased significantly compared with the IOP at 5 min after the injection (*p* < 0.01). it was in normal range but still higher than baseline (*p* < 0.01; Fig. 1). The CD at 2 min, 5 min, and 30 min after injection were 2604.55 ± 381.42 cells/mm^2^, 2637.16 ± 284.61 cells/mm^2^, and 2623.82 ± 275.34 cells/mm^2^, respectively. There was a slight increase, but it was not significant (all, *p* > 0.05). The CV of the endothelium increased 2 min after injection, but it was also not significant (*p* = 0.8). After injection, the 6A of the endothelium and CCT also did not show any significant change (*p* > 0.05; Table 5). In 37 patients who didn’t have cataract surgery, the parameters except IOP did not show a significant difference over time (Table 6).

**Table 5:** Characteristics of Endothelial Cells and IOP in Patients Undergoing Intravitreal Bevacizumab Injection (mean ± SD)

	Baseline (*n* = 42)	After 2 min	After 5 min	After 30 min
IOP(mmHg)^a^	11.48 ± 2.22	49.71 ± 10.73	37.64 ± 11.68	14.88 ± 4.77
CD(cells/mm^2^)^a^	2605.01 ± 399.08	2604.55 ± 381.42	2637.16 ± 284.61	2623.82 ± 275.34
CV ^a^	62.65 ± 32.90	89.01 ± 74.71	74.61 ± 31.14	76.81 ± 35.77
6A(%)^a^	39.95 ± 10.77	36.05 ± 14.94	38.02 ± 10.95	36.81 ± 2.44
CCT(μm)^a^	507.00 ± 32.54	505.12 ± 27.66	503.95 ± 30.46	513.19 ± 28.17

*After* after injection, *IOP* intraocular pressure, *CD* corneal density, *CV* coefficient of variation, *6A* hexagonality, *CCT* central corneal thickness, *Baseline* before injection

^a^IOP significant increased in a stepwise manner (all, *p* < 0.01). After 30 min, it normalized but still higher than baseline (*p* < 0.01). But there were no significant change in CD, CV, 6A, or CCT according to IOP over time than baseline (all, *p* > 0.05) by one-way repeated measures ANOVA, Bonferroni correction

**Table 6:** Characteristics of Endothelial Cells and IOP in Patients of phakic eyes Undergoing Intravitreal Bevacizumab Injection (mean ± SD)

	Baseline (*n* = 37)	After 2 min	After 5 min	After 30 min
IOP(mmHg)^a^	11.57 ± 2.27	50.19 ± 10.68	38.35 ± 11.66	14.97 ± 5.00
CD(cells/mm^2^)^a^	2673.41 ± 370.58	2673.88 ± 333.57	2656.46 ± 293.05	2674.32 ± 247.79
CV^a^	64.05 ± 31.80	89.38 ± 78.67	75.51 ± 32.09	76.54 ± 37.58
6A(%)^a^	40.30 ± 10.29	37.16 ± 15.45	38.76 ± 9.78	38.54 ± 11.85
CCT(μm)^a^	510.21 ± 34.78	505.88 ± 29.56	505.76 ± 31.54	516.00 ± 29.35

*After* after injection, *IOP* intraocular pressure, *CD* corneal density, *CV* coefficient of variation, *6A* hexagonality, *CCT* central corneal thickness; Baseline: before injection

^a^IOP significant increased in a stepwise manner (all, *p* < 0.01). After 30 min, it normalized but still higher than baseline (*p* < 0.01). But there were no significant change in CD, CV, 6A, or CCT according to IOP over time than baseline (all, p > 0.05) by one-way repeated measures ANOVA, Bonferroni correction

**Fig. 2:**
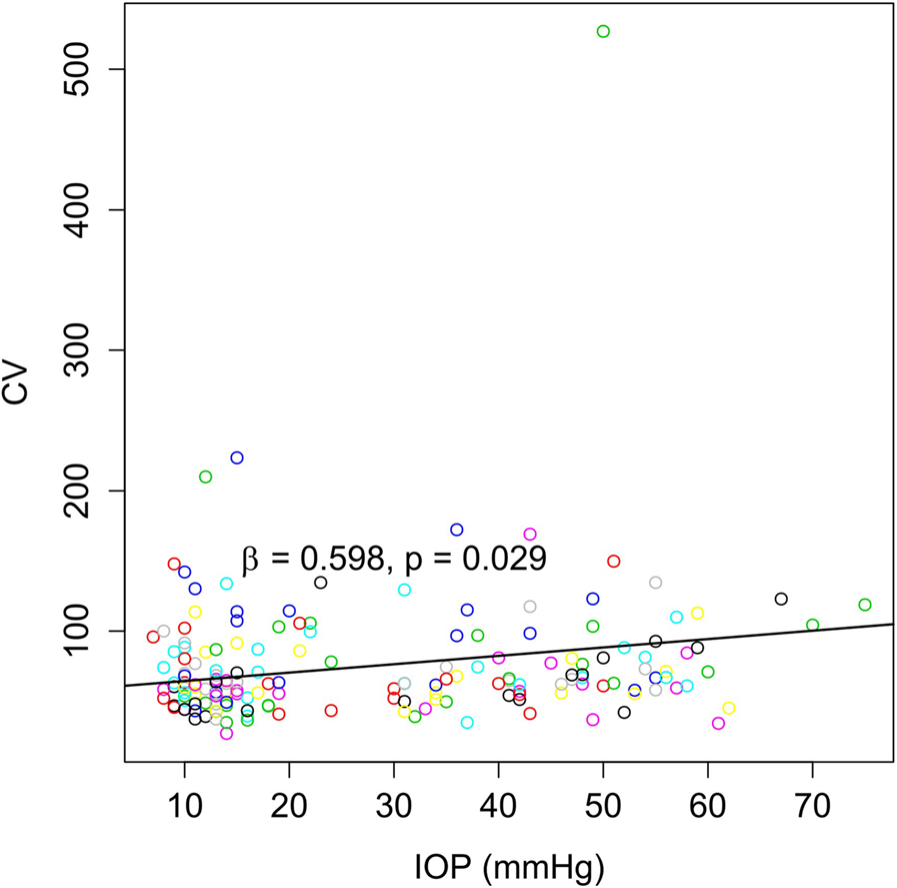
Coefficient of variation of the cell area (CV) changes at different intraocular pressure (IOP) after intravitreal bevacizumab injection. Linear mixed model with *p* < 0.05. CV increased significantly with increased intraocular pressure

We thought that there is a correlation between each repeated measurement in the same subject according to the change of IOP after injection. So, we calibrate it and reanalyzed, as a result, only the CV of the endothelium significantly increased with increasing IOP (ß = 0.60, *p* = 0.03; Fig. 2). The other parameters, including endothelial CD, 6A of the endothelium, and CCT did not show any significant correlation with changes of IOP (*p* = 0.79, 0.21, and 0.08, respectively; Table 7). The same analysis was performed for 37 phakic eyes. As above, only the CV of the endothelium significantly increased with increasing IOP (ß = 0.61, *p* = 0.04). The other parameters did not show significant results as above (Table 8). One week after injection, there was no sign of inflammation or any other side effects in all 42 eyes.

**Table 7:** Relationships between IOP and Endothelial Cells or CCT Undergoing Intravitreal Bevacizumab Injection in all patients (*n* = 42)

Y^b^	ß1 (IOP)	*p*-value^a^	ß2 (time)	*p*-value^a^	ß3 (IOP^a^ time)	*p*-value^a^	Y-intercept
CD(cells/mm^2^)	−0.40	0.79	0.49	0.92	−0.02	0.95	2632.05
CV	0.60	0.03	0.95	0.25	−0.04	0.34	58.42
6A(%)	−0.08	0.21	−0.15	0.44	0.01	0.71	40.50
CCT(μm)	−0.13	0.08	−0.22	0.36	0.03	0.05	508.24

*CD* cell density, *CV* coefficient of variation, *6A* hexagonality, *CCT* central corneal thickness, *IOP* intraocular pressure

^a^Mixed lineal model

^b^Y = ß1 × IOP + ß2 × time + ß3 × IOP × time + Y-intercept

**Table 8:** Relationships between IOP and Endothelial Cells or CCT Undergoing Intravitreal Bevacizumab Injection in patients excluding pseudophakic eyes (*n* = 37)

Y^b^	ß1 (IOP)	*p*-value^a^	ß2 (time)	*p*-value^a^	ß3 (IOP^a^ time)	*p*-value^a^	Y-intercept
CD(cells/mm^2^)	−0.33	0.83	2.08	0.68	−0.16	0.61	2695.86
CV	0.61	0.04	0.92	0.31	−0.04	0.46	57.76
6A(%)	−0.04	0.48	−0.03	0.88	−0.01	0.96	40.36
CCT(μm)	−0.13	0.11	−0.26	0.32	0.03	0.04	509.31

*CD* cell density, *CV* coefficient of variation, *6A* hexagonality, *CCT* central corneal thickness, *IOP* intraocular pressure

^a^Mixed lineal model

^b^Y = ß1 × IOP + ß2 × time + ß3 × IOP × time + Y-intercept

## Discussion

Corneal endothelial cells play an important role in maintaining the structural integrity, transparency, and dehydrated state of the cornea. Once damaged, they rarely regenerate [1, 2]. In young adults, the mean endothelial CD is 3500 cells/mm^2^ and the CV of the endothelium is 0.25 in normal subjects. The normal range of 6A of the endothelium is 60~ 80%, which would be reduced if endothelial cells were damaged [10].

Many studies have reported factors affecting the corneal endothelium. It is generally known that the endothelial CD gradually decreases with age. But whether the polymegathism and polymorphism increase with age to cover the emerging endothelium defect is controversial [11–13]. Endothelial cell changes could result from systemic diseases such as DM [14–17]. Laule et al. [18] reported that endothelial cells are annually reduced by approximately 0.5% during physiological aging. Koo et al. [19] reported that the CCT thickens and the endothelial CD and 6A decrease during DM longer than 10 years. However, in the present study, there was no significant difference in any parameters in patients with and without DM.

Photorefractive keratectomy or laser assisted in situ keratomileusis have been reported to be safe for endothelial cells [20], but even simple cataract surgery like PE&PCL can damage these cells although it is usually not big impact [21, 22]. The more difficult and complex the surgery, the higher the risk of endothelial cell damage [23]. In cases of glaucoma patients, the endothelial CD decreases as the duration of topical eye drop use for lowering IOP lengthens [24], and there is a possibility that other ophthalmic or systemic diseases could also negatively affect corneal endothelial cells. Comparing the groups according a history of cataract surgery, CD of pseudophakic eyes were significantly lower than that of phakic eyes. The patients who underwent cataract surgery were statistically significantly older than the phakic patients, so no conclusion can be drawn if age or the procedure (not “surgery”) affected the cell density.

IOP is also an important factor that affects corneal endothelial cells. The degree of corneal hydration is mainly maintained by the endothelial pump, but it is also determined by the barrier of epithelium, evaporation on the corneal surface, IOP, and swelling pressure(SP). SP of corneal stroma is caused by the action of the anion of the side chain of glycosaminoglycan such as keratin sulfate and chondroitin sulfate present in the substrate to push each other. In normal corneal stroma without edema, SP is about 55 mmHg. The anion present in the glycosaminoglycan of corneal stroma attracts the cationic sodium ion and absorbs water, it is called as imbibition pressure (IP). Among these factors including SP, IP, and IOP, there is a relation of ‘IP = IOP – SP’. Depending on the degree of IOP and SP, IP is determined, resulting in corneal edema and endothelial damage [25].

It is reported that when an AACG attack occurs within 2 days, the endothelial CD does not significantly decrease, but if the duration is over 3 days, the endothelial CD decreases. Furthermore, the longer the duration of the attack, the greater the decrease of endothelial CD [5]. High IOP itself can affect endothelial cells by three-mechanisms, direct mechanical damage, impaired endothelial pump, and ischemic, oxidative stress. Direct mechanical damage affects corneal endothelial deformation and resulting compression and stretch on the endothelium [26]. Long-term deformation can cause a significant effect on the endothelial function and it may result in endothelial dysfunction. High IOP may affect the function of endothelial pump and induced corneal edema [27, 28]. And it also reduces intraocular blood flow, induces hypoxia and oxidative stress and as a result, could damage endothelial cells.

We measured the endothelial CD, shape, and CCT at baseline, and 2 min, 5 min, and 30 min after injection to determine if there were any changes in endothelial cells during the period of increased IOP. As reported in previous studies, IOP first increased, then decreased with time. Among other endothelial cell parameters, only the CV of the endothelium significantly increased with increased IOP. We performed analyses again removing the pseudophakic eyes and found that only the CV significantly changed, as above. In this study, an acute increase of IOP caused endothelial cells to stretch through direct mechanical damage as described above, we supposed, it could explain the significant correlation between CV and IOP. This is also consistent because this parameter is the most sensitive measure of cornea endothelial dysfunction [11] (Tables 6 and 7; Fig. 2). The mean IOP at 2 min after injection was 49.71 ± 10.73 mmHg which is lower than normal stromal SP. And because IOP of most eyes returned to a normal range, it could not cause a long-term endothelial cell pump dysfunction or ischemic change, we supposed.

There were some limitations in this study. First, the sample population was small and the duration of DM, age, and sex were not considered. Further studies should be performed with larger sample populations, which could better characterize changing patterns and the mechanism of action of corneal endothelial cells during and after acute IOP changes by intravitreal bevacizumab injection. Second, a non-contact tonometer (CT-80; Topcon) was used because of the possibility of infection instead of a Goldmann applanation tonometer that produces the most accurate results. However, we used the mean value of three measurements that can be accepted as a reliable result. Third, this study reflected only the initial 30 min, but it is enough time for the IOP to return to normal levels. Although the IOP after 30 min was still higher than baseline, it is no longer outside the normal range after it has normalized, and this has already been shown in some previous studies [6, 29]. So it is sufficient time to analyze corneal endothelial cell changes according to the change of IOP after injection.

## Conclusions

Despite its limitations, this study is the first to observe initial momentary change of corneal endothelial cells by specular microscopy immediately after IOP elevation by intravitreal bevacizumab injection. When bevacizumab is intravitreally injected, an acute increase of IOP occurs, but after 30 min, the IOP normalized. During this process, endothelial cells may receive momentary compression and stretch pressure, but it is tolerable and has no harmful effects on the corneal endothelium.

## Abbreviations

6A: Hexagonality
CCT: Central corneal thickness
CD: Cell density
CV: Coefficient of variation of the cell area
DM: Diabetes mellitus
IOP: Intraocular pressure
IP: Imbibition pressure
PE&PCL: Phacoemulsification and posterior chamber lens implantation
SP: Swelling pressure
